# Hydro-Salpingitis. A Case Cured with Gelseminum

**Published:** 1893-04

**Authors:** J. H. Allen

**Affiliations:** Logansport, Ind,


### HYDRO-SALPINGITIS. A CASE CURED WITH GELSEMINUM.

J. H. ALLEN, M. D., LOGANSPORT, IND.

CASE.—Mrs. E., aged twenty-seven, light blonde, above medium height, and weight about one hundred and forty, came to me one year ago for an examination and treatment for a large growth in the left abdominal region. Pregnancy had been diagnosed, with advice to rest as much as possible, especially during the menstrual period, which function had kept up with clock-like regularity every month. During the inter-menstrual period she suffered very little pain with exception of an occasional dragging or bearing-down feeling in the region of the uterus. As the usual period of gestation had passed she became very anxious to know what the trouble was. After examining her case carefully I made a diagnosis of some tubular trouble, and that of cystic nature and in all probability hydro-salpingitis.

Though it is hard to diagnose these cases, yet by taking into consideration that the case had a gonorrhœal history, together with the location of tumor, the consistency of the walls of the growth and by the aid of percussion and the bimanual examination, together with the large amount of serum that passed at the close of each menstrual period I made it pretty conculsive. The tumor was then the size of a child's head or larger, mobile and fluctuating, the os was patulous, the genital organs showed marked signs of pregnancy and in an advanced stage, morning sickness had continued all through her illness, the breasts had increased in size and were fully as large as in the eighth month. She was now suffering considerable pain in the region of the left ovary, which was probably due to pressure or distention caused by the accumulated fluid, as it was worse just before the menses and better after the profuse flow of serum that came on at the close of the menstrual period.

I called in an eminent surgeon from a distant city in consultation, who advised an operation for removal of the ovary as well as the diseased tube. I did not agree with him as to his mode of procedure, and my patient would not listen to it for a moment. I therefore concluded to try my remedies and await results. On examination of the symptomatology of her case the following symptoms were noted: A feeling of fullness and heaviness in the uterine region, spasmodic, cramp-like pains during the menses, sharp pains running from uterus to back and hips. A languid aching in back and hips a day or so before the menses, great weakness and loss of power in the lower extremities, very little pain after the menses begin; she says that her pains resemble false labor pains, when she gets tired she has a lump in the throat which she cannot swallow. After the menses suffers with pains in back of head and spine, pains running up the back of the neck, with a feeling of tightness in the brain, she is irritable and easily angered. There is considerable fever in the afternoons and evenings accompanied with twitching of the muscles. The menses last eight days, for the first three days they seem quite natural, but during the rest of the flow it is very light-colored and resembles serum.

Gelseminum was given in the one thousandth potency, which relieved her menstrual pains, and as she continued to improve and feel better generally I did not change the prescription. In ten days after receiving the remedy the flow of serum increased to almost a pint per day for a week, when it gradually decreased and finally ceased entirely. The next menstrual period only lasted five days, she experiencing very little pain and no return of the flow of serum. In all six powders of the remedy were given during the period of two months, usually a powder was prepared in water and given every hour until she found relief when it was discontinued. Since then she has received no medicine, though it is nine months since I discharged the case. At the end of the third month all symptoms of the tumor had disappeared, and the menses resumed the normal again, and she has been in perfect health since.

